# Efficacy and safety of the CAPTEM regimen in patients with advanced adrenocortical carcinoma

**DOI:** 10.1530/EC-26-0136

**Published:** 2026-10-09

**Authors:** Barbara Ziółkowska, Agnieszka Kotecka-Blicharz, Daria Handkiewicz-Junak, Kornelia Hasse-Lazar, Tomasz Gawlik, Adam Idasiak, Aleksandra Krzywon, Rafał Suwiński

**Affiliations:** ^1^ 2nd Department of Radiotherapy and Chemotherapy, Maria Sklodowska-Curie National Research Institute of Oncology, Gliwice Branch, Gliwice, Poland; ^2^ Department of Nuclear Medicine and Endocrine Oncology, Maria Sklodowska-Curie National Research Institute of Oncology, Gliwice, Poland; ^3^ Department of Biostatistics and Bioinformatics, Maria Sklodowska-Curie National Research Institute of Oncology, Gliwice Branch, Gliwice, Poland

**Keywords:** adrenocortical carcinoma, gemcitabine, temozolomide, CAPTEM

## Abstract

**Purpose:**

The purpose of this study was to evaluate the activity and tolerability of the CAPTEM regimen (capecitabine + temozolomide) in patients with advanced, heavily pretreated adrenocortical carcinoma (ACC).

**Methods:**

This is a retrospective analysis of 15 patients with metastatic ACC who received at least two lines of systemic therapy, including the CAPTEM regimen, between 2017 and 2022. Clinical characteristics, treatment outcomes (PFS and OS), and toxicities were collected and analysed. Treatment response was assessed using the RECIST 1.1 criteria. Survival outcomes were estimated using the Kaplan–Meier method.

**Results:**

Among the 15 patients, CAPTEM was administered in the first-line therapy in three patients (20%), in the second-line therapy in eight patients (53.3%), and in further lines in the remaining cases. The overall disease control rate was 26.6% (all stable disease). The median progression-free survival (PFS) was 3 months (95% CI: 2–6), and the median overall survival (OS) was 8 months (95% CI: 6–13). Patients who achieved disease stabilization had improved outcomes (mPFS: 9.5 months; mOS: 16.5 months) compared with those with progression (mPFS: 2 months; mOS: 7 months). Treatment-related adverse events occurred in 86.7% of patients, mostly mild; grade 3 toxicity was observed in one patient.

**Conclusion:**

The CAPTEM regimen demonstrated modest activity and acceptable toxicity in a heavily pretreated ACC population. While not recommended as a standard treatment, CAPTEM may be considered in select patients, particularly those who achieve disease stabilization. Further prospective studies are needed to better define its role and identify predictive biomarkers.

## Introduction

Adrenocortical carcinoma (ACC) is a rare and aggressive neoplasm with an estimated incidence of 0.5–2 new cases per million people per year (1). For locally advanced ACC, the treatment of choice is surgical resection followed by adjuvant mitotane therapy at high risk of relapse defined as positive margin, large volume, high grade, and ruptured capsule (2).

Standard treatment for recurrent, locally advanced, or metastatic ACC is mitotane. In addition, patients with rapid progression and high tumour volume should be offered chemotherapy. The activity of a triple-agent regimen with doxorubicin, cisplatin, and etoposide with or without mitotane (EDP-M or EDP, respectively) had been proven in the FIRM-ACT phase 3 trial (3, 4). The use of the triple-agent combination statistically prolonged progression-free survival (PFS) by 2.9 months compared with streptozocin, although there were no significant differences in overall survival (OS), presumably due to the crossover and presence of mitotane in both arms. Currently, it remains the standard of care for advanced ACC.

Data supporting second-line and subsequent systemic therapies in advanced adrenocortical carcinoma (ACC) remain limited and largely derived from small prospective trials and retrospective analyses. No standard treatment has been established in this setting, and therapeutic decisions are often individualized based on patient performance status, tumour burden, and prior treatments.

Cytotoxic chemotherapy regimens have shown modest activity. In a phase II study, gemcitabine combined with metronomic capecitabine demonstrated disease stabilization in 46% of patients at four months, with a median progression-free survival (PFS) of 5.3 months and overall survival (OS) of 9.8 months (5). Although these results suggest some clinical benefits, responses are rarely durable. Immunotherapy has also been investigated as a potential treatment option. Two phase II trials evaluated the efficacy of pembrolizumab in advanced ACC. In one study including 16 patients, the primary end point of non-progression at 27 weeks was achieved in 36% of patients, with partial responses observed in 14% and stable disease in 50% of cases (6). In a larger cohort of 39 patients, the objective response rate was 23%, although the median PFS remained limited at 2.1 months, despite a relatively long median OS of 24.9 months (7). These findings indicate that a subset of patients may derive benefit from immune checkpoint inhibition, but predictive biomarkers are lacking. Alkylating agents, such as temozolomide, have also been explored. In a retrospective study of 28 heavily pretreated patients, temozolomide monotherapy resulted in tumour shrinkage in 21% of cases; however, this did not translate into improved survival outcomes, with limited PFS and OS (8). The activity of temozolomide may be influenced by the methylation status of the MGMT gene, although this association has not been clearly established in ACC. More recently, targeted therapies have shown encouraging preliminary results. Cabozantinib, a multityrosine kinase inhibitor targeting c-MET, VEGFR2, and RET, demonstrated promising efficacy in a phase II trial, with 72% of patients achieving progression-free survival at four months and a median PFS of 7.2 months (9). Nevertheless, these findings require validation in larger cohorts. Given the absence of a clearly effective standard in the second-line setting and beyond, there is a strong rationale to investigate alternative therapeutic strategies.

The CAPTEM regimen is a combination of capecitabine and temozolomide that has shown activity in numerous neuroendocrine tumours (10). Temozolomide is an oral alkylating agent that showed a cytotoxic and cytostatic effect through apoptosis, inhibition of cell growth, and cell cycle arrest in ACC cells *in vitro* (11). An association was also observed between the cytotoxic effect of temozolomide and the epigenetic silencing of MGMT (O6-methylguanine-DNA methyltransferase), which corresponds to efficacy in patients with glioblastoma (12). Capecitabine is an antimetabolite that incorporates 5-fluorodeoxyuridine triphosphate into the DNA, leading to attenuation of the repair activity of MGMT through inhibition of thymidylate synthase and reduction in thymidine levels (13).

The purpose of the present study was to evaluate the activity and tolerance of the CAPTEM regimen in patients with heavily pretreated ACC.

## Materials and methods

Data from all 22 patients with adrenocortical carcinoma (ACC) treated at a Polish oncological institution (Maria Sklodowska-Curie National Research Institute of Oncology, Gliwice Branch) between 2017 and 2022 were retrospectively collected and reviewed. All patients received at least one line of chemotherapy in the metastatic setting. Seven patients who did not receive the CAPTEM regimen were excluded from the analysis. Ultimately, 15 patients with complete clinical data who had received at least two lines of systemic treatment were included. 

The following data were collected for each patient: age, sex, histological features, ECOG performance status, treatment administered, treatment outcomes (progression-free survival (PFS) and overall survival (OS)), and treatment-related toxicity. Tumour stage was assessed according to the modified ENSAT (mENSAT) classification.

Patients received the CAPTEM regimen consisting of capecitabine at a dosage of 600–750 mg/m^2^ administered orally twice daily on days 1–14 and temozolomide at a dosage of 150–200 mg/m^2^ administered orally in two divided doses daily on days 10–14 of a 28-day cycle. Dose modifications were allowed based on treatment tolerance and clinical judgement.

The duration of the CAPTEM therapy was not predefined and depended on treatment tolerance, response, and patient preference. Tumour response was assessed according to the Response Evaluation Criteria in Solid Tumors (RECIST), version 1.1.

### Statistical analysis

Continuous data were categorized according to median values and interquartile ranges (IQRs, 25–75%). Categorical variables are shown as frequencies and percentages. OS and PFS were estimated using the Kaplan–Meier method, and 95% CIs for the survival curves were calculated. PFS was defined as the time from the onset of systemic therapy until disease progression as confirmed by clinical examination, radiological evaluation, death, or the last date of patient follow-up. OS was calculated as the time from the start of treatment until death from any cause or the last date of patient follow-up. All computational analyses were performed in the R environment for statistical computing, version 4.0.1, ‘See Things Now’, released on 6 June 2020 (R Foundation for Statistical Computing, Austria, http://www.r-project.org).

## Results

### Patients’ characteristics

Table 1 summarizes the patients’ baseline characteristics. The study cohort included eight males and seven females, with a median age of 47 years (range: 19–76). The majority of patients had a good performance status (ECOG PS 0–1, 86.6%) and hormonally active tumours (73.3%). Adrenalectomy was performed in 13 patients (86.7%).

Eight patients (53.3%) received adjuvant mitotane therapy. Data on plasma mitotane concentrations were limited and available only for a small subset of patients (*n* = 3), in whom therapeutic levels were achieved. All of them eventually developed disease progression and were referred for systemic treatment. Progression occurred both during active mitotane therapy and after its discontinuation. The median time to progression was approximately 8.9 months (range: 2.0–49.6 months). Most patients progressed during ongoing treatment, and two of them experienced progression after mitotane discontinuation, with a median interval of 11.2 months (range: 7.1–15.3 months).

**Table 1:** Patient characteristics. Values are presented as *n* (%).

Characteristics	Values
Number of patients	15
Age, years (median)	47 (19–76)
Sex	
Males	8 (53%)
Females	7 (47%)
Histological characteristics	
Ki-67 < 10%	7 (47%)
Ki-67 < 10–30%	4 (27%)
Ki-67 > 30%	4 (27%)
Weiss score	6.9 (5–9)
Secreting at diagnosis	11 (73.3%)
Androgen excess	4 (26.6%)
Cortisol excess	8 (53.3%)
Non-secreting tumours	4 (26.6%)
Primary surgery	13 (86.7%)
Adjuvant mitotane	8 (53.3%)
First-line treatment in advanced disease	
EDP	12 (80%)
CAPTEM	3 (20%)
CAPTEM in advanced disease	
First line	3 (20%)
Second line	8 (53.3%)
Third line	3 (20%)
Fourth line	1 (6.7%)
Mitotane in advanced disease	12 (80%)
ECOG PS	
0	13 (86.6%)
>1	2 (13.4%)
mENSAT	
IVA	10 (66.6%)
IVB	3 (20%)
IVC	2 (13.3%)

EDP, etoposide + doxorubicin + cisplatin; ECOG PS, Eastern Cooperative Oncology Group performance status; mENSAT, modified ENSAT staging system; IVA, metastases in two organs, including lymph nodes; IVB, metastases in three organs; IVC, metastases in more than three organs.

Mitotane was administered according to standard dosing schedules, with dose adjustments based on tolerability. Due to the retrospective nature of the study, detailed information regarding treatment exposure, including precise dosing and potential mitotane-free intervals, was incomplete.

According to the modified ENSAT (mENSAT) classification, 10 patients presented with stage IVA disease (66.6%), three with stage IVB (20%), and two with stage IVC (13.3%).

### Treatment administered

In the first-line setting, EDP combination regimen (etoposide + cisplatin + doxorubicin) was administered in 12 patients and three patients received CAPTEM (capecitabine + temozolomide). The CAPTEM regimen in the first-line setting was given to patients with ECOG PS > 1 and impaired renal function. In 12 cases, mitotane was added to cytotoxic therapy. The median PFS for first-line EDP was 3.5 months, and the median OS was 18 months.

Taking into account the line of treatment, CAPTEM was administered in the first-line therapy in three patients (20%), in the second-line setting in eight patients (53.3%), in the third-line setting in three patients (20%), and in the fourth-line setting in one patient (6.7%). Twelve patients continued concomitant mitotane therapy, with therapeutic concentrations achieved in nine of them (75%) (Table 2). The remaining three patients did not receive mitotane due to unacceptable toxicity that had developed during prior adjuvant treatment. The mitotane dosing schedule was individualized according to patient tolerance and clinical condition. In the analysed cohort, both rapid- and slow-dose escalation regimens were used in accordance with the ENSAT/ESE guidelines for adrenocortical carcinoma.

**Table 2:** Treatment response according to patient characteristics.

No.	RECIST 1.1	CAPTEM line of treatment	Mitotane treatment	Mitotane therapeutic level	ENSAT stage	Ki-67	Weiss score	Metastatic sites	Tumour burden (≤5 vs > 5 metastases)
1	PD	3	Yes	Yes	II	≤10%	7	Lung	≤5
2	SD	4	No	N/A	IV	≤10%	7	Local recurrence, liver	>5
3	PD	2	Yes	Unknown	IV	≤10%	Unknown	Liver	≤5
4	PD	2	Yes	Yes	II	>30%	6	Lymph nodes, liver, local recurrence	>5
5	PD	2	Yes	Yes	III	10–30%	9	Lymph nodes, liver, local recurrence, others	>5
6	PD	2	Yes	Yes	III	≤10%	8	Lymph nodes, local recurrence	≤5
7	PD	2	No	N/A	III	10–30%	5	Liver, others	≤5
8	PD	1	No	N/A	III	>30%	Unknown	Lymph nodes	≤5
9	PD	3	Yes	Yes	II	≤10%	Unknown	Others	>5
10	PD	3	Yes	Yes	II	10–30%	6	Liver, bone, local recurrence, others	>5
11	PD	2	Yes	Yes	IV	>30%	Unknown	Lung	≤5
12	PD	2	Yes	Unknown	IV	10–30%	7	Lung, lymph nodes	>5
13	SD	2	Yes	Yes	IV	>30%	Unknown	Lung, lymph nodes, liver	>5
14	SD	1	Yes	Yes	II	≤10%	7	Lung, bone, local recurrence	>5
15	SD	1	Yes	No	I	≤10%	Unknown	Local recurrence	≤5

RECIST 1.1, Response Evaluation Criteria in Solid Tumors, version 1.1; PD, progressive disease; SD, stable disease.

The median number of cycles of the CAPTEM regimen was 5.2 (range: 2–21). Overall, four patients (26.6%) achieved stable disease according to the RECIST 1.1 criteria, whereas 11 patients (73.3%) experienced disease progression. Among patients treated with CAPTEM in the first-line setting, stable disease was observed in one patient, while two patients progressed. In the second-line setting, disease stabilization occurred in two out of eight patients, with progression in six. In the third-line setting, one out of three patients achieved stable disease and two experienced progression, whereas the single patient treated in the fourth-line setting had disease progression. When analysed according to treatment line, the median progression-free survival (PFS) was 6 months in the first-line setting, 2.5 months in the second-line setting, 2 months in the third-line setting, and 18 months in the fourth-line setting. The median overall survival (OS) was 13, 7.5, and 6 months for the first-, second-, and third-line treatments, respectively; OS was not considered informative in the single fourth-line case. Among patients who progressed, five had ECOG performance status 0–1 and received further systemic therapy: two patients received EDP and three received pembrolizumab.

Patients who received CAPTEM, regardless of the line of therapy, achieved a median PFS of 3 months (95% CI: 2–6) (Fig. 1A) and a median OS of 8 months (95% CI: 6–13) (Fig. 1B). When stratifying patients according to therapy and response to the therapy, the median PFS was 9.5 months and the median OS 16.5 months, in patients who achieved stabilization, while in those who progressed, the median PFS was 2 months (Fig. 2A) and the median OS 7 months (Fig. 2B).

**Figure 1:**
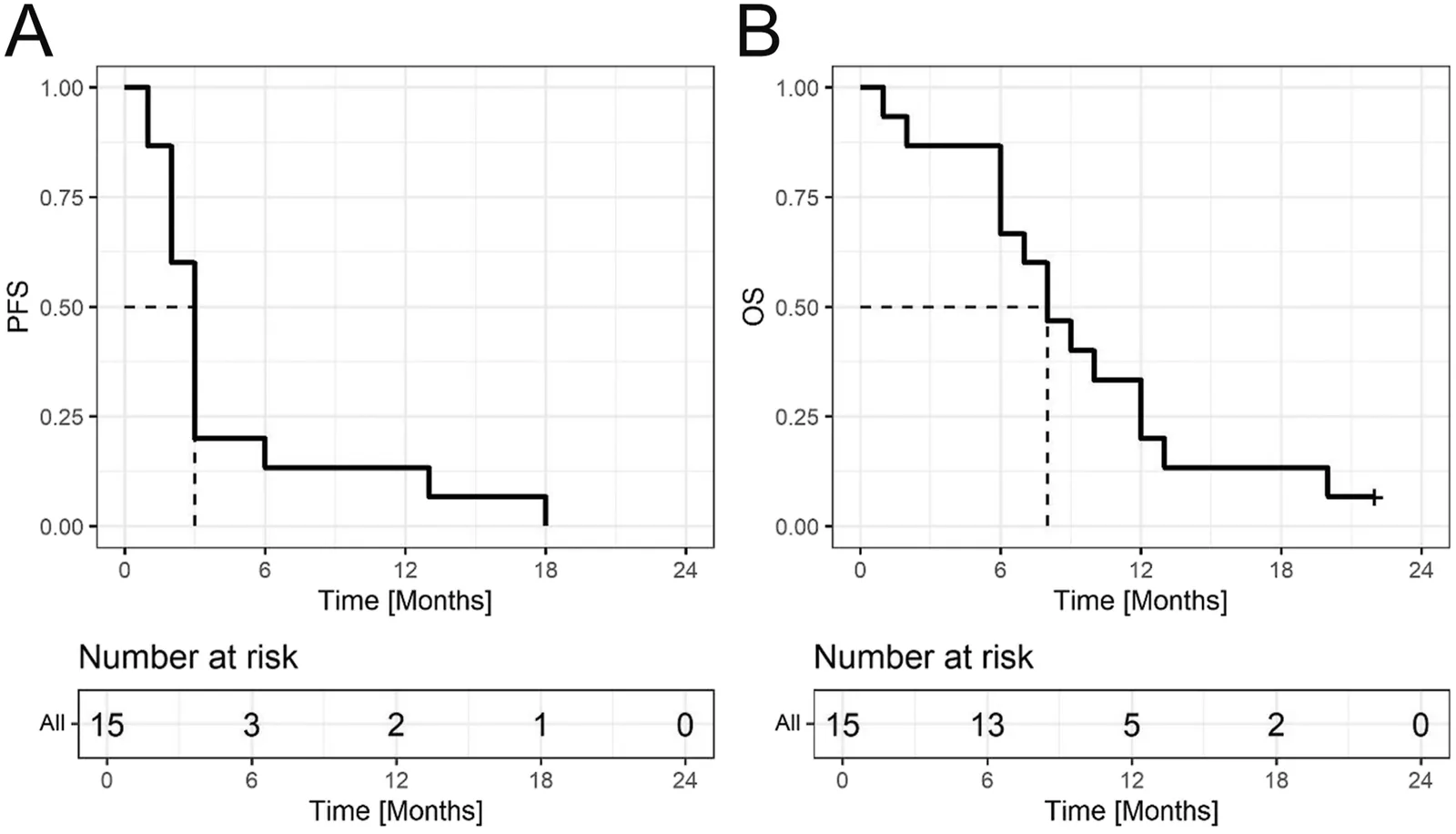
(A) Progression-free survival (PFS) of patients treated with CAPTEM regardless of the line of therapy; (B) overall survival (OS) of patients treated with CAPTEM regardless of the line of therapy.

**Figure 2:**
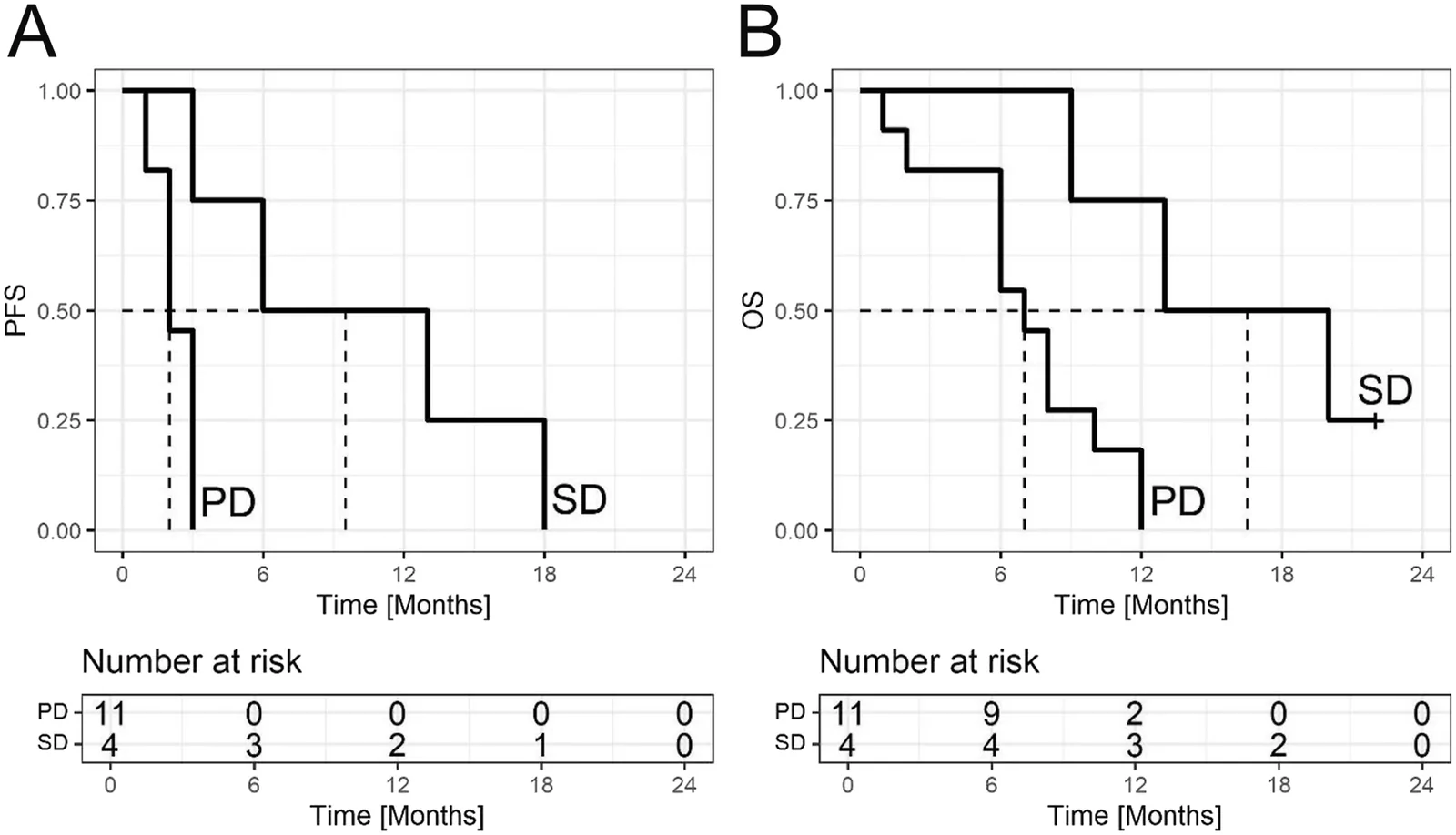
(A) Progression-free survival (PFS) stratified according to response to CAPTEM therapy; (B) overall survival (OS) stratified according to response to CAPTEM therapy.

### Treatment toxicity

Treatment-related toxicities are summarized in Table 3. Blood chemistry and clinical examination were performed before each cycle of chemotherapy. Adverse events of any grade, according to the Common Terminology Criteria for Adverse Events, version 4.0 (CTCAE v.4.0), occurred in 13 patients (86.7%). The most frequent side effect was anaemia, occurring in 12 patients, and one patient developed grade 3 (G3) anaemia. Both neutropenia and thrombocytopenia were observed in three patients. Five patients developed liver toxicity of G1. Fatigue and nausea were observed in six and one patient, respectively. There was only one G3 toxicity and no G4. Three patients required dose reduction due to the toxicity.

**Table 3:** CAPTEM toxicity (*n* = 30 (100%)). Adverse events according to the Common Terminology Criteria for Adverse Events, version 4.0 (CTCAE v.4.0).

	All grades	G1	G2	G3
Anaemia	12 (40%)	5 (16.7%)	6 (20%)	1 (3.3%)
Neutropenia	3 (10%)	2 (6.7%)	1 (3.3%)	0 (0%)
Thrombocytopenia	3 (10%)	3 (10%)	0 (0%)	0 (0%)
Liver toxicity	5 (16.7%)	5 (16.7%)	0 (0%)	0 (0%)
Fatigue	6 (20%)	6 (20%)	0 (0%)	0 (0%)
Nausea	1 (3.3%)	1 (3.3%)	0 (0%)	0 (0%)

G, grade; G1, mild; G2, moderate; G3, severe.

## Discussion

ACC is poorly responsive to systemic treatments, and heavily pretreated patients have a very short life expectancy. Given the rarity of ACC, most of the data on therapeutic approaches come from case reports or retrospective studies. Prospective phase 3 randomized trials still remain an unmet need. In cases of metastatic or inoperable ACC with low tumour burden and slow progression, defined as a disease-free interval greater than 1 year after initial radical therapy, mitotane in monotherapy is a valuable option. In the German study (14), patients treated with mitotane only achieved a mPFS of 4.1 months and a mOS of 18.5 months. In cases with a high tumour burden and an aggressive course of disease, a triple-agent regimen with doxorubicin, cisplatin, and etoposide, with or without mitotane (EDP-M or EDP, respectively), remains the standard of care (4). Nevertheless, over 50% of patients will progress within six months after initiating the treatment.

In this report, we present the results of systemic treatment for heavily pretreated ACC patients. In the first-line setting, 12 out of 15 patients received the standard EDP-M regimen and the other three received the CAPTEM regimen. The mPFS in the EDP group was 3.5 months and the mOS was 18 months, which is consistent with data from the FIRM-ACT trial (4). There are very limited data on the second and subsequent lines of chemotherapy for ACC, but patients with good ECOG PS might be offered second-line treatment upon progression.

When comparing both treatment approaches, a direct comparison between the EDP-M and CAPTEM groups should be interpreted with caution due to substantial differences in treatment lines and baseline patient characteristics. Nevertheless, the median progression-free survival was 3.5 months in patients treated with first-line EDP-M and 3 months in the CAPTEM group overall, suggesting broadly comparable but limited efficacy of both regimens in this advanced ACC population.

In our study group, 12 (80%) patients who progressed on EDP and were subsequently treated with CAPTEM in the second or further lines of therapy achieved a median PFS of 3 months (95% CI: 2–6) and a median OS of 8 months (95% CI: 6–13). When analysis was restricted to this subgroup of 12 patients, results remained consistent with the overall CAPTEM cohort. In radiological evaluation, none of these patients achieved partial or complete response according to the RECIST 1.1 criteria, while stable disease was observed in four cases (33%). In this subgroup, the median PFS was longer in patients achieving disease stabilization compared with those with progression (9.5 vs 2 months), as well as median OS (16.5 vs 7 months). Although adverse events occurred in 86.7% of patients, they were mostly mild and manageable.

The moderately active CAPGEM (capecitabine + gemcitabine) regimen had shown efficacy in one prospective trial (5) and in a few retrospective studies and case reports (15, 16, 17). In the Sperone *et al.* trial (5), the mPFS was 5.3 months and the mDSS (median disease-specific survival) was 9.8 months, which was superior to the mPFS obtained in the Henning *et al.* (18) case series (mPFS: 3 months and mDSS: 10 months). Temozolomide in monotherapy also showed a modest activity (8, 19) with a partial response of 21.4%, but a poor mPFS and mOS of 3.5 and 7.2 months, respectively. It is, thus, not recommended as a standard of care.

Recent advances in immunotherapy and molecularly targeted therapies have opened new options for ACC. Although the role of immune checkpoint inhibitors remains unclear, the results from two phase 2 trials showed modest effectiveness of pembrolizumab (6, 7). The use of pembrolizumab resulted in non-progression at 27 weeks in 36% of patients in one study (6), while an objective response rate of 23% was reported in another trial (7). In our cohort, three patients received pembrolizumab after progression on CAPTEM; however, due to the very small sample size, no meaningful conclusions regarding efficacy could be drawn. Nevertheless, these findings support the limited but potential activity of immunotherapy in selected ACC patients.

Cabozantinib, a multityrosine kinase inhibitor targeting RET, c-Met, AXL, and vascular endothelial growth factor (VEGF), is approved for advanced kidney cancer, liver cancer after previous sorafenib, and medullary thyroid carcinoma. In the phase 2 trial, the primary endpoint – progression-free survival at four months – was achieved in 72% of patients (9). These data are consistent with results from a retrospective study in which PFS >16 weeks was observed in 50% of patients (20). Although no patients in our cohort received cabozantinib, the available evidence suggests that tyrosine kinase inhibitors may represent a promising therapeutic option in advanced ACC.

This study provides the first data on the efficacy of the CAPTEM regimen in advanced ACC. This study has several limitations typical for retrospective research with a small number of patients. Although CAPTEM showed modest efficacy in this cohort, its role in the therapeutic landscape of ACC remains limited and should not be recommended as a standard therapeutic option for ACC unless the potential biomarkers indicate the subgroup that can benefit.

## Declaration of interest

The authors declare that there is no conflict of interest that could be perceived as prejudicing the impartiality of the work reported.

## Funding

This research did not receive any specific grant from any funding agency in the public, commercial, or not-for-profit sector.

## Author contribution statement

BZ and AK-B conceived the study and performed data acquisition and curation; BZ, AK-B, and AK performed data analysis; BZ wrote the original draft. BZ, AK-B, AK, DH-J, AI, RS, and TG, KH-L performed review and editing.

## Ethics approval and consent to participate

This is a retrospective non-interventional study, and all data were collected as a part of routine clinical practice for diagnosis and treatment according to national regulations. The study protocol was approved by the Institutional Review Board of Maria Sklodowska-Curie National Research Institute of Oncology (ethical board approval number: 46/2025). Administrative permissions were obtained from the directors of the oncology departments and information centres to access the raw data from the hospital medical records. The Institutional Review Board of the Maria Sklodowska-Curie National Research Institute of Oncology, Gliwice Branch, has waived the need to obtain informed consent due to the retrospective nature of the study.
